# CLASSIE teaching – using virtual reality to incorporate medical ethics into clinical decision making

**DOI:** 10.1186/s12909-020-02217-y

**Published:** 2020-09-23

**Authors:** Adrienne Torda

**Affiliations:** Faculty of Medicine, UNSW Sydney, Kensington, NSW 2052 Australia

## Abstract

**Background:**

Teaching medical ethics (ME) in the clinical environment is often difficult, uncalibrated and medical students get variable exposure to skilled educators. Explicit discussion of ethical dimensions of patient management is often neglected, as clinical teachers may feel inadequately skilled to do this.

**Methods:**

We developed a suite of online modules. Each consisted of a clinical scenario filmed using virtual reality (VR) technology, linked to an adaptive, interactive, online tutorial which explicitly discussed the relevant ethical issues and guidelines. These were embedded in clinical placements of students to encourage the transfer of knowledge from these modules to clinical skill competency.

We conducted a pilot study to evaluate these modules which examined student engagement, knowledge gains (self-perceived and measured) and user experience. We also reviewed reflections to assess the incorporation of these modules and transfer of knowledge into the clinical learning and skill development of the students.

**Results:**

Engagement and self-perceived knowledge gains were extremely high. Students found these modules realistic, interesting and helpful. The measured knowledge gains (module exit quiz) were moderate. User experience was positive overall, although students were intolerant of any technical glitches. There was mixed feedback on whether the VR aspect of the clinical scenarios added value. Student reflections showed high level incorporation of these modules into clinical practice of the students and evidence of knowledge transfer (level 3 Kirkpatrick model of evaluation) in over ¾ of students.

**Conclusions:**

This study showed that the use VR clinical scenarios combined with interactive online learning modules resulted in demonstrable high-level student engagement and learning gains in medical ethics and transfer of knowledge to clinical application. It standardised and ensured the student experience of high-quality educational deliverables in clinical years of medical education. This use of VR and online technology can be adapted for use in many areas of the medical curricula where we need to ensure the delivery of well calibrated, high quality, educational deliverables at scale for students.

## Background

Medical ethics (ME) is quite a controversial and variably taught component of medical curricula in most countries [1–4]. There is ongoing discussion about the varying importance of teaching ethical knowledge, ethical awareness and skills in medical decision making [5–7]. The bioethicist Judith Andre wrote that teaching ethics is “fundamentally an act of hope” [8]. We usually have no idea whether such has an impact on the future behaviour of doctors [9, 10]. In fact, it has been recognised that often external factors within health care system and the ‘hidden curriculum’ [11] act against the successful implementation of ME education [5, 12, 13]. Yet the presence of ME as a core component of medical curricula, even if we cannot quantitate its impact or value, is consistent with the goals and commitment we make to the importance of this component of the curriculum [1, 7, 10]. The presence of ME teaching is now mandated for accreditation of medical programs in many countries including Australia, the United States and the United Kingdom [14–16].

Although curricula vary, the bulk of ME teaching is generally done in the clinical context where it makes more sense to students and moral competency is embedded in clinical decision making. In this environment, competencies and milestones can be referred to and assessed, as components of ‘Entrustable Professional Activities’ [17]. One problem with this, is that much of this teaching and assessment is done by clinical educators and supervisors who are not specifically skilled in ME education. They are generally clinicians with variable ability themselves to identify and discuss ethical issues in the clinical context and often have variable ability to support the development of knowledge and skills of the students in this competency. Students also have very different and personalised learning journeys in the clinical environment and may encounter very variable opportunities in relation to teaching opportunities for ME. Yet students are all assessed at the same level, usually established by National guidelines. To overcome this issue of variable ME teaching in the clinical environment, we developed a suite of online modules, each of which contained a clinical scenario linked to an adaptive online tutorial. These were called ‘Clinical adaptive student studies in Ethics’ or ‘CLASSIE’ modules. These were developed to complement and be done simultaneously with the appropriate clinical attachment. Although a formal needs assessment was not conducted, informally this need was clearly identified from student feedback. The students wanted high quality, standardised teaching in ME.

## Methods

Clinical scenarios were filmed using a 360 camera, so that the scenarios could be viewed using 3-dimensional technology to create a clinical virtual reality (VR) environment for the viewer. The rationale behind this, was that the student could ‘enter’ this environment, allowing visual and aural immersion which would allow full focus on the scenario and stimulate appropriate emotional, as well as intellectual responses from the students. The aim was to simulate the real clinical experience as much as possible. Each scenario covered a specific topic related to the student’s clinical attachment. These scenarios were selected as they were identified as the top ten ‘problematic’ areas for senior students on clinical rotations in feedback from previous years. They are also included in the issue list identified by Donaldson et al. [6]. Initially 10 were developed, covering topics in Medicine, Surgery, Paediatrics, Obstetrics, Primary care and Psychiatry, these are detailed in Table 1. For example, the emergency department scenario showed an end of life discussion with an elderly woman and her family, and highlighted decision complexities. In this scenario the husband and the daughter of the patient had very different opinions about the management boundaries they thought were appropriate for their wife/mother, who had late stage dementia and was requiring ventilatory support. Not only did the entire scenario play out for the student (as if they were in the room), they could also listen to each of the stakeholders discussing individually why they felt the way they did. The maximum duration of a scenario was 8 min.

**Table 1:** Scenario content for Classie Medical Ethics modules

Course and Theme	Module scenario content
Medicine – Advance care planning	A woman with dementia and pneumonia is admitted to hospital without any clear management plan or advance care directive and with different opinions from members of her family.
Medicine – Ethical issues in organ transplantation	A young man has been on a waiting list for a renal transplant for some time and decides to travel back to Iran, where his grandmother lives, as he may be able to purchase a kidney.
Surgery – Consent and religious objection	A 15 year old boy presents with severe gastro-intestinal bleeding. His parents will not let him have a blood transfusion as they are Jehovah’s witnesses.
Paediatrics – Complementary medicine, consent and minors	A couple with a toddler with leukemia want to stop conventional therapy and opt for treatment with naturopathy only
Emergency medicine – Do not resuscitate orders	A man is brought into the Emergency department after a cardiac arrest. He has suffered hypoxic brain injury and there is little hope of functional recovery. They need to make a decision about what to do if he has a cardiac arrest
Psychiatry – Consent and vulnerable patients	A diabetic patient with schizophrenia needs a surgical amputation of her foot. The surgeon needs to determine if she has capacity to consent.
Psychiatry – The impaired practitioner	Two junior doctors have noticed that one of their medical colleagues is acting irrationally, making mistakes and intermittently has come to work smelling of alchohol
Primary care – Management of mature minors	A 15 year old girl presents to her primary care physician asking for a prescription for a contraceptive pill. Her doctor needs to determine how to manage this
Obstetrics – Termination of pregnancy	A young woman who has just found out that she is pregnant presents to her obstetrician asking for a termination of pregnancy
Obstetrics – In Vitro Fertilisation (IVF)	A wealthy 51 year old woman asks her obstetrician for IVF. Her obstetrician needs to work through a number of ethical issues including her age

Each clinical scenario was linked to an interactive adaptive tutorial created using Smart Sparrow software, which delivered an interactive tutorial about this topic. Each module had specific learning objectives relevant to the ethics theme. Content for these modules was created using a team of educational developers which included medical students. Each tutorial included an identical entry quiz and an identical exit quiz, so that we could extract analytics about knowledge gains, time spent in module etc. There were also questions and activities throughout each module. Students were required to complete each module to mastery to obtain a certificate of completion which could be used as evidence of learning in this capability. They were also requested to complete a reflection about what they learned.

These modules were evaluated in a pilot study. This was conducted on a single day using final year medical student participants who completed a minimum of three online modules, then an anonymous online questionnaire (52 participants). A subset also participated in a focus group (20 participants). A mixed method approach was used to evaluate these modules. We examined student engagement, knowledge gains (self-perceived and measured) and user experience of VR learning. Subsequent thematic analysis of student reflections was also performed. All analyses of the questionnaire responses were descriptive, either categorical using Likert scale responses or open-ended responses. Analytics of time spent and learning gains were extracted from the Smart Sparrow software. After submission, 20 student reflections were also reviewed to further assess the impact of these modules on student learning about the clinical application of the Classie modules.

## Results

### Student engagement

**Fig. 1:**
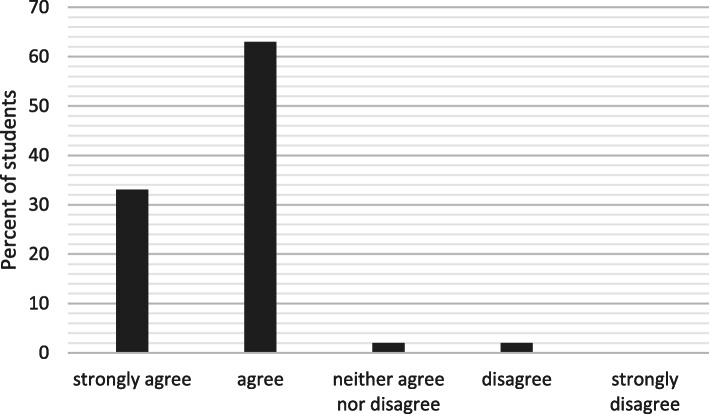
The learning modules were engaging: This figure illustrates the percentage of students agreeing with each response from ‘strongly agree’ to ‘strongly disagree’ in relation to the statement ‘the learning modules were engaging’

Students found these modules highly engaging and interesting as shown in Fig. 1. They also stated that they were realistic and related to their clinical experience. Approximately 40% said that these modules exposed them to scenarios that they hadn’t come across clinically. They felt that this was a ‘great initiative’ and asked for more modules to be created.

### Knowledge gains

**Fig. 2:**
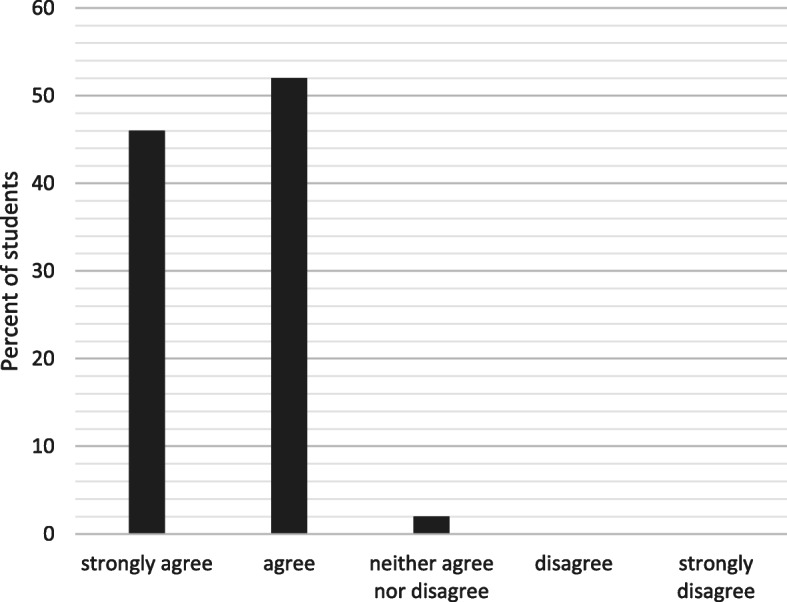
The learning module increased my knowledge in this area: This figure illustrates the percentage of students agreeing with each response from ‘strongly agree’ to ‘strongly disagree’ in relation to the statement ‘the learning modules increased my knowledge in this area’

**Fig. 3:**
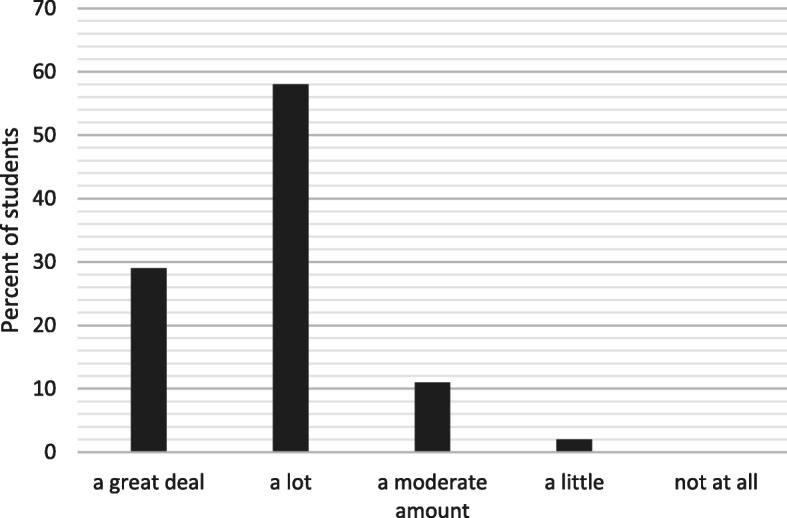
Did the content in the modules help you understand issues that affect clinical decision making?: This figure illustrates the percentage of students with each response from ‘a great deal’ to ‘not at all’ in relation to the question ‘Did the content in the modules help you understand issues that affect clinical decision making?’

Self-perception of knowledge gains was high amongst students. Most students (> 90%) either agreed or strongly agreed that these modules had increased their knowledge (Fig. 2) and helped their understanding of the ethical issues that affect clinical decision making (Fig. 3). Knowledge gains as measured using quiz results were more moderate. Approximately 10% of students received full marks on entry quiz. The average entry mark was 50% and between 22 and 50% received full marks on their first try of the exit quiz (depending on the module), with an average mark 60% on first attempt. Most students took 2–3 attempts at the exit quiz (to get 100%).

### User experience of VR learning

In the pilot study, the user experience of VR learning was mixed. In the questionnaire 50% of students felt that the 3-D experience enhanced the clinical scenario videos, 25% were neutral and 25% did not. This mixed response was one of the main themes further explored in the focus groups. The themes that further emerged as reasons for this were:

1. Frustration with download speed. Despite all students participating in a university computer lab, there were problems with stuttered download of videos.
2. The desire by a small number of students to speed up scenario videos, so that they could watch them faster. Some students also wanted to have a transcript of the scenarios that they could read, rather than spend time to watch them.

### Reflection and transfer of learning to clinical application

The transfer of reflective learning from the ME modules into improved clinical skill was assessed by review of student reflections on changes in clinical skills and experience. Forty one students wrote reflections that showed evidence of transfer of knowledge acquired from the ME modules to clinical skills. The students’ reflections demonstrated that they had integrated content from the online ME with their clinical skill development. Students not only stated that the scenarios were relevant, realistic and hugely value-added to their clinical learning, they used specific examples to illustrate how they had changed either their behaviour or understanding of a clinical situation. Students were able to utilitize and apply what they had learnt from the ME modules to real clinical contexts. They also stated that the virtual scenarios captured the complexity of ethical issues in real clinical scenarios well and hence helped them with clinical decision making.

### Some excerpts are shown below

*• I had been in the exact scenario as Psychiatry 1 - Schizophrenia. In that session, we did not address the ethical issues with the patient, and the doctors did not explain them either. This scenario helped me understand and then be able to deal with such a situation in real life.*

• *While I thought that I had understood the guardianship and idea of ‘person responsible’, in reality that can be quite a nuanced concept, and this module was a fantastic learning experience for situations in which the answers aren’t black or white. It helped me have the skill to handle a similar clinical situation.*

• *The ethics modules were particularly helpful in giving me a ethical framework to navigate some of the more difficult clinical situations that I then came across in hospital and will encounter in the future as a doctor.*

• *Completing these ethics modules was a unique experience in simulating the reality of ethical dilemmas in clinical practise. Typically, ethics teaching involves going through philosophical ethical principles that, while interesting, can seem quite abstracted and removed from the day-to-day reality of medicine. However, with the incorporation of 3D virtual reality video in these modules, I was able to see how ethical principles play out in real life before my very eyes. This helped me acquire the skills to understand these issues when they came up in the hospital.*

## Discussion

ME is a core component of most medical programs in many countries and is required for program accreditation in a great number, including Australia, New Zealand, USA, Canada and the UK. Despite this, there remains no consensus in the literature or in practice about the main goal or best methods of teaching medical ethics [18]. Hence the delivery of this component of medical curricula is often uneven and can be problematic, as clinicians vary in their ability to discuss the ethical aspects of clinical scenarios. This study provided evidence that the use of CLASSIE modules were a very useful teaching tool for teaching ME. They were extremely well received and had a positive impact on student learning in this competency. The students were highly engaged by this learning activity, and the reflections showed that this learning was being well integrated with real clinical experiences of the students.

Although, the data could only be assessed using descriptive statistics, they showed a very high level of positive user experience, self-perceived learning gains and moderate objectively assessed learning gains. If we relate this evaluation to the Kirkpatrick model [19], we not only show learning at level one (reaction) and two (learning), analysis of student reflections shows definitive evidence of transfer of this knowledge (level three) in over ¾ of students. In their reflections, the students specifically described how these modules enabled them to identify areas of personal improvement and consciously apply their new knowledge to clinical situations, consistent with intensive reflective learning on the non-reflective to reflective learning continuum [20]. This is particularly important, as skill development and application in relation to competency in medical ethics is notoriously difficult to assess. Using evidence of reflective learning to evaluate the transfer of knowledge into clinical practice is well supported by the medical education literature, particularly that looking at development of ‘professionally competent clinical practice’, [21] professional identity [22–24] and personal growth [25]. This is based on the belief that our ‘professional identities’ are constantly being re-shaped by our experiences and environment [26, 27]. Educators can track this development (to some extent) by examining the narratives (or reflections) of students. Reflective writing is generally considered in itself to be a means of developing ethical mindfulness [28], critical thinking skills [29, 30], clinical reasoning [31] and the ability to manage complex and ambiguous situations in medicine [32], such as those described in the Classie modules. Even this assessment of knowledge transfer however is limited, in that we cannot determine if the students’ capacity for decision making or action in a clinical situation was also changed.

In clinical settings, ME may be taught variably, hence this approach allows the delivery of high quality, calibrated educational deliverable to all medical students, independent of their clinical placements and tutors. The use of mastery learning in the quiz is consistent with an increasingly popular paradigm for medical education. The basic principle is that educational excellence is expected, with minimal variation in measured outcomes, but students will take varying amounts of time to get to that point [33, 34]. In addition to filling a potential teaching gap, the fact that the CLASSIE modules can be accessed flexibly in time and place, was also positively received by students. This approach to the use of internet learning to fill gaps in clinical exposure or experience can be usefully adapted for teaching in any context where either we have inconsistent teaching skills (such as ME) or even difficulties with student access (such as obstetrics or paediatrics) [35]. It will also allow medical programs to share the best teaching materials, eventually saving on resources and ensuring best practice. The specific negative feedback in the pilot helped us to explain the context and improve on technical issues. The VR video format definitely added to the immersive and emotional nature of the doctor-patient encounter videos and made it similar to being in the room during the consultation. According to adult learning theory, it is thought that emotion and cognition are interdependent and that emotions are important for learning and problem solving, as they enable more flexible and adaptive thinking [36]. This modern approach to instructional design goes beyond the pure cognitive aspects of learning and incorporates the ‘affective’ aspects. ‘How has VR value-added in this space?’ is a difficult question to answer. The simulated environment in general, is aimed at replicating real life, and in doing so, lets the learner act (or at least think about how they would act) as in real life. VR involves a totally immersive world and one of the benefits is that is makes access to the clinical experience simply and flexibly available [37]. There is evidence in addition to this study that demonstrates that VR and the immersion it offers adds to effective experiential learning [38]. That fact that students were highly engaged and enjoyed this as a learning experience, also adds to its learning value [36]. These experiences should be integrated with, and complement everyday clinical practice [39]. In extreme times when clinical placements become difficult, such as during the COVID-19 pandemic, VR can become a very useful resource. The role of VR in anatomy teaching [40] and assisting with teaching technical skills [41, 42] has been well evaluated [39]. If VR is not available, these scenarios could definitely be created in other ways, dependent on the available resources. These modules could be created using normal video equipment, with volunteer actors, with live simulated patients or alternatively, if this is not feasible, they could use animations or just a description of the scenario to prompt the interactive online learning activities.

Although it is evident that technology won’t solve all our problems as educators and we need to be aware of the limitations [43], technology such as this, can dramatically assist in providing some calibration of learning opportunities for medical students. This study also revealed that although we like to encourage a deeper level of learning in all our students, some are still quite strategic and will try to skim through learning activities, particularly online ones. Trying to change this approach is still a challenge for medical educators but explaining the rationale for why we do things and providing high quality educational deliverables definitely helps. In general, the students were very appreciative of efforts such as these to improve learning resources.

## Conclusions

The creation and use of online learning modules, such as the CLASSIE modules can complement and integrate with the clinical experiences of medical students. In this study we were able to show that a high quality, well calibrated educational resource could be used to produce high level learning in a complex competency, such as medical ethics. Future work will involve an evaluation of the specific contribution of the VR technology over other technological approaches to teaching ME and an expansion of this approach to other competencies that may be difficult to teach in a calibrated way during clinical placements. This approach to the use of technology to support student learning, can be used to complement any areas of medical curricula where opportunities may be scarce or variable.

## Data Availability

The datasets used and/or analysed during the current study are available from the corresponding author on reasonable request.

## Abbreviations

Classie: Clinical adaptive student studies in Ethics
IVF: In-vitro fertilisation
ME: Medical ethics
VR: Virtual reality

## References

[1] Carrese JA, Malek J, Watson K, Lehmann LS, Green MJ, McCullough LB, Geller G, Braddock CH, Doukas DJ (2015). The essential role of medical ethics education in achieving professionalism: the Romanell report. Acad Med.

[2] Kavas MV, Ulman YI, Demir F, et al. The state of ethics education at medical schools in Turkey: taking stock and looking forward. BMC Med Educ. 2020;20(1):162. Published 2020 May 24. 10.1186/s12909-020-02058-9. 10.1186/s12909-020-02058-9 PMC7245803 32448274

[3] Doukas DJ, McCullough LB, Wear S (2012). Project to rebalance and integrate medical education (PRIME) investigators. Perspective: medical education in medical ethics and humanities as the foundation for developing medical professionalism. Acad Med.

[4] Torda A, Mangos JG. Medical ethics education in Australian and New Zealand (ANZ) medical schools: a mixed methods study to review how medical ethics is taught in ANZ medical programs. Int J Ethics Educ. 2020; 10.1007/s40889-020-00097-w.

[5] Eckles RE, Meslin EM, Gaffney M, Helft PR (2005). Medical ethics education: where are we? Where should we be going?. Rev Acad Med.

[6] Donaldson T, Fistein E, Dunn M (2010). Case-based seminars in medical ethics education: How medical students define and discuss moral problems. J Med Ethics.

[7] Giubilini A, Milnes S, Savulescu J (2016). The medical ethics curriculum in medical schools: Present and future. J Clin Ethics.

[8] Andre J. Bioethics as Practice. North Carolina: United States of America: The University of North Carolina Press, Chapel Hill; 2003.

[9] Doukas DJ, Kirch D, Brigham TP, Barzansky BM, Wear S, Carrese JA, Fins JJ, Lederer SE (2015). Transforming educational accountability in medical ethics and humanities education toward professionalism. Acad Med.

[10] Campbell AV, Chin J, Voo TC (2007). How can we know that ethics education produces ethical doctors?. Med Teach.

[11] Rajput V, Mookerjee A, Cagande C, 2017, ‘The Contemporary Hidden Curriculum in Medical Education’, Med Ed Publish, 6, [3], 41. 10.15694/mep.2017.000155.

[12] Doukas DJ, McCullough LB, Wear S, Lehmann LS, Nixon LL, Carrese J, Shapiro JF, Green MJ, Kirch DG (2013). For the project to rebalance and integrate medical education (PRIME) investigators the challenge of promoting professionalism through medical ethics and humanities education. Acad Med.

[13] Stirrat GM (2015). Reflections on learning and teaching medical ethics in UK medical schools. J Med Ethics.

[14] Liaison Committee on Medical Education. Functions and structure of a medical school. Standards for accreditation of medical education programs leading to the MD degree. Standard 3.5. 2017 http://lcme.org/publications/ Accessed July 30, 2020.

[15] Australian Medical Council. Standards for Assessment and Accreditation of Medical Schools by the Australian Medical Council 2012. https://www.amc.org.au/accreditation-and-recognition/accreditation-standards-and-procedures/. Accessed 3 Sept 2020.

[16] General Medical Council. Generic Professional capabilities framework. https://www.gmc-uk.org/-/media/documents/generic-professional-capabilitiesframework--0817_pdf-70417127.pdf. Accessed 3 Sept 2020.

[17] Lomis K (2017). Implementing an Entrustable professional activities framework in undergraduate medical education: early lessons from the AAMC Core Entrustrable professional activities for entering residency pilot. Acad Med.

[18] Goldie J (2000). Review of ethics curricula in undergraduate medical education. Med Ed.

[19] Kirkpatrick Model. Four Levels of Learning Evaluation. Educational Technology. https://educationaltechnology.net/kirkpatrick-model-four-levels-learningevaluation/. Accessed 3 Sept 2020.

[20] Peltier JW, Hay A, Drago W (2005). The reflective learning continuum: reflecting on reflection. J Mark Educ.

[21] Wald HS, Reis SP. Beyond the margins: reflective writing and development of reflective capacity in medical education. J Gen Intern Med. 2010;25(7):746–9. 10.1007/s11606-010-1347-4. Epub 2010 Apr 21. PMID: 20407840; PMCID: PMC2881974. 10.1007/s11606-010-1347-4 PMC2881974 20407840

[22] Wald HS, Borkan JM, Taylor JS, Anthony D, Reis SP. Fostering and evaluating reflective capacity in medical education: developing the REFLECT rubric for assessing reflective writing [published correction appears in Acad Med. 2012;87(3):355]. Acad Med 2012;87(1):41–50. 10.1097/ACM.0b013e31823b55fa. 10.1097/ACM.0b013e31823b55fa 22104060

[23] Wald HS (2015). Professional identity (trans) formation in medical education: reflection, relationship, resilience. Acad Med.

[24] Wald H, Weiss B. 'Making it “More Real”: Using Personal Narrative in Faculty Feedback to a Medical Student’s Reflective Writing – An Illustrative Exemplar', MedEdPublish. 2018;7(3):33. 10.15694/mep.2018.0000171.1. 10.15694/mep.2018.0000171.1 PMC10701800 38074617

[25] Song P, Stewart R (2012). Reflective writing in medical education. Med Teach.

[26] Monrouxe LV (2010). Identity, identification and medical education: why should we care?. Med Educ.

[27] Frost HD, Regehr G (2013). “I AM a doctor”: negotiating the discourses of standardization and diversity in professional identity construction. Acad Med.

[28] Guillemin M, Gillam L (2015). Emotions, narratives, and ethical mindfulness. Acad Med.

[29] Mamede S, Schmidt HG, Penaforte JC (2008). Effects of reflective practice on the accuracy of medical diagnoses. Med Educ.

[30] Driessen E, van Tartwijk J, Dornan T (2008). The self critical doctor: helping students become more reflective. BMJ..

[31] Plack MM, Greenberg L (2005). The reflective practitioner: reaching for excellence in practice. Pediatrics..

[32] Eichbaum QG (2014). Thinking about thinking and emotion: the metacognitive approach to the medical humanities that integrates the humanities with the basic and clinical sciences. Perm J.

[33] Cook DA, Brydges R, Zendejas B (2013). Mastery learning for health professionals using technology-enhanced simulation: a systematic review and meta-analysis. Acad Med.

[34] McGaghie WC (2015). Mastery learning. Acad Med.

[35] Wong G, Greenhalgh T, Pawson R (2010). Internet-based medical education: a realist review of what works, for whom and in what circumstances. BMC Med Educ.

[36] Um E, Plass J, Hayward E, Homer B (2012). Emotional design in multimedia learning. J Educ Psychol.

[37] Pottle J. Virtual reality and the transformation of medical education. Future Health J. 2019;6(3):181–5. 10.7861/fhj.2019-0036. PMID: 31660522; PMCID: PMC6798020. 10.7861/fhj.2019-0036 PMC6798020 31660522

[38] Makowski D, Sperduti M, Nicolas S, Piolino P (2017). ‘Being there’ and remembering it: presence improves memory encoding. Conscious Cogn.

[39] Samadbeik M, Yaaghobi D, Bastani P, Abhari S, Rezaee R, Garavand A. The Applications of Virtual Reality Technology in Medical Groups Teaching. J Adv Med Educ Prof. 2018;6(3):123–129. PMID: 30013996; PMCID: PMC6039818. PMC6039818 30013996

[40] Moro C, Štromberga Z, Raikos A, Stirling A (2017). The effectiveness of virtual and augmented reality in health sciences and medical anatomy. Anat Sci Educ.

[41] Gallagher AG, Ritter EM, Champion H, Higgins G, Fried MP, Moses G, Smith CD, Satava RM. Virtual reality simulation for the operating room: proficiency-based training as a paradigm shift in surgical skills training. Ann Surg. 2005;241(2):364–72. 10.1097/01.sla.0000151982.85062.80. PMID: 15650649; PMCID: PMC1356924. 10.1097/01.sla.0000151982.85062.80 PMC1356924 15650649

[42] Schroedl CJ, Corbridge TC, Cohen ER, Fakhran SS, Schimmel D, McGaghie WC (2012). Use of simulation-based education to improve resident learning and patient care in the medical intensive care unit: a randomized trial. J Crit Care.

[43] Sklar D (2018). Can words on the screen replace the face in the classroom? Using the internet to revolutionize medical education. Acad Med.

